# The involvement of the circFOXM1–miR–432–Gα12 axis in glioma cell proliferation and aggressiveness

**DOI:** 10.1038/s41420-021-00782-9

**Published:** 2022-01-10

**Authors:** Yong Gong, Shuai Zhang, HongXin Wang, Yunfeng Huang, Xing Fu, Peng Xiang, Tianyu Fan

**Affiliations:** The Affiliated Changsha Central Hospital,Department of Neurosurgery, Hengyang Medical School, University of South China, No.161, Shaoshan South Road, Changsha, Hunan 410004 China

**Keywords:** Oncogenes, CNS cancer

## Abstract

Accumulating evidence indicates that circFOXM1 (Hsa_circ_0025033) is highly expressed in several cancers; however, the function of circFOXM1 in glioma and the molecular mechanism have not been well explored. In the present study, we found that expression of circFOXM1 was upregulated in both glioma tissues and cell lines. In addition, circFOXM1 knockdown suppressed glioma-cell proliferation, activated apoptosis in vitro, and repressed tumour growth in vivo. Moreover, we clarified that circFOXM1 binds with miR-432, which was downregulated in glioma cells. Furthermore, we indicated that Gα12, a direct target of miR-432, was highly expressed in glioma cells, and Gα12 silencing might limit the progression of glioma. Rescue assays indicated that Gα12 reversed the inhibitory effect of circFOXM1 silencing on glioma-cell tumorigenesis. In conclusion, circFOXM1 acts as a sponge of miR-432 to promote the proliferation and aggressiveness of glioma cells through the Gα12 signalling pathway.

## Introduction

Glioma is one of the most common and aggressive central-system tumours and has become the primary cause of the loss of life and independence, conveying a heavy burden [1, 2]. At present, despite technical advancements in invasive surgical resection and postoperative radiotherapy or chemotherapy, the overall 5-year survival rate of glioblastoma patients remains poor [3, 4]. Therefore, it is urgent to explore the in-depth mechanism to lay the foundation for the diagnosis and treatment of glioma.

Circular RNAs (circRNAs) are a type of endogenous noncoding RNA that was first discovered from exons or introns through backsplicing [5, 6]. To date, circRNAs have been widely identified in various transcriptomes [7, 8] and exhibit low-level expression, however, their expression is higher than their linear counterparts [9]. Previous studies demonstrated that circRNAs play a crucial role in cancer progression and have become biomarkers for early cancer diagnosis [10–12]. The mechanism of circRNAs includes acting as miRNA sponges or competing endogenous RNAs, binding and secluding proteins, and regulating splicing [13].

CircFOXM1 (Hsa_circ_0025033) has been verified to be highly expressed in cancers, such as non-small-cell lung carcinoma, papillary thyroid carcinoma, and hepatocellular carcinoma. It serves as an oncogene to regulate cell migration, invasion, and tumour growth [14–16]. However, the function of circFOXM1 in glioma and the molecular mechanism are less well understood.

Gα12, a member of the heterotrimeric guanine nucleotide-binding protein (G protein), has been detected in various tissues, and Gα12 might associate with diverse proteins [17, 18]. A previous study showed that Gα12 has potential functions in stress-fibre formation, cytoskeletal rearrangement, and cell-growth regulation [19]. Gα12 has been revealed to contribute to several cancers [20, 21]. However, whether a correlation exists between Gα12 and glioma is unknown.

In the present study, we demonstrated that expression of circFOXM1 is upregulated in both glioma tissues and cell lines. In addition, circFOXM1 knockdown suppressed cell proliferation and activated glioma apoptosis in vitro and inhibited tumour growth in vivo. Moreover, we clarified that circFOXM1 binds to miR-432 using RIP and luciferase-reporter assays, and miR-432 is downregulated in glioma cells. The miR-432 downstream gene Gα12 is highly expressed in glioma cells, indicating that Gα12 silencing might limit the progression of glioma. In the rescue experiment, upregulation of Gα12 compensated for the inhibitory effect of circFOXM1 silencing on glioma-cell growth. Our study confirmed a regulatory mechanism of the circFOXM1–miR-432–Gα12 axis in glioma, suggesting that targeting circFOXM1 may represent a promising approach for future glioma treatment.

## Results

### Identification of circFOXM1 in glioma cells

In a previous study, FOXM1 was reported to be closely associated with tumorigenesis, and upregulation of FOXM1 in most human cancers conveyed poor prognosis in patients. Therefore, we analysed the FOXM1 gene in the UCSC database and discovered that the exons encode 13 circRNAs (Fig. S1A). Then, 12 primers were established to measure the expression of these 13 circRNAs in glioma-cell lines and tissues. The results of semiquantitative PCR indicated that circ-0025033 from primer 1 (P1) and primer 12 (P12) amplification exhibited strong positive expression, demonstrating differential expression from other cancers in a previous study (Fig. S1B, C). Therefore, we referred to circ_0025033 as circFOXM1 in glioma.

**Fig. 1:**
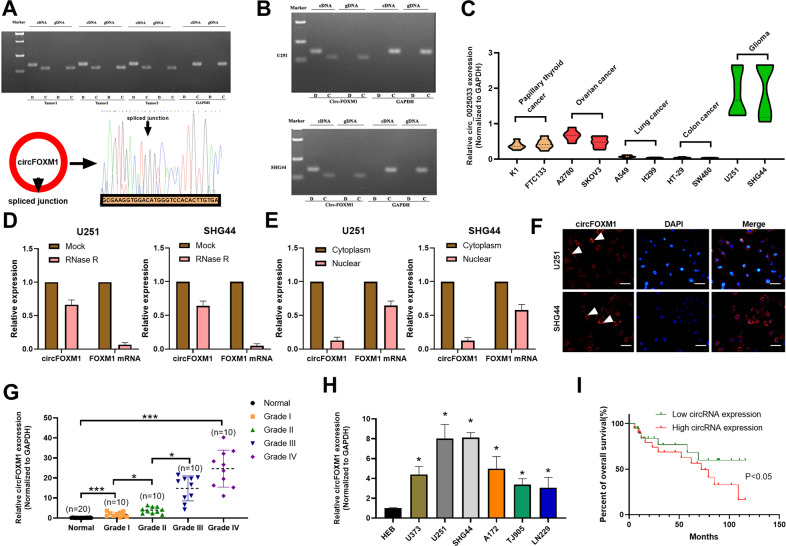
Identification of circFOXM1 in glioma cells. **A** Two pairs of primers were designed to detect the presence of circFOXM1 in 3 glioma tissues (D represents the reverse primer and C represents the forward primer). **B** PCR assay to detect the expression of circFOXM1 in two glioma-cell lines. **C** qRT-PCR to detect the expression of circFOXM1 in different cancer-cell lines. **D** Expression of circFOXM1 in glioma cells via qRT-PCR when RNase R was used. **E** Expression of circFOXM1 in the cytoplasm and nucleus of glioma cells via qRT-PCR. **F** circFOXM1 localization in glioma cells by immunofluorescence. **G** Relative expression of circFOXM1 in glioma tissues of different grades. **H** Relative expression of circFOXM1 in different glioma cell lines. **I** Survival curve of glioma patients with high expression of circFOXM1 and low expression of circFOXM1 (*P < 0.05, ***P < 0.001).

Afterward, the expression of circFOXM1 in glioma tissues donated from 3 glioma patients was assessed via PCR. The results showed that circFOXM1 was remarkably upregulated in both glioma tissues (Fig. 1A) and in U251 and SHG44 glioma-cell lines (Fig. 1B). qRT-PCR results revealed that expression of circ_0025033 in glioma-cell lines was higher than that in other tumour cell lines, such as papillary thyroid carcinoma, ovarian cancer, lung cancer, and colon cancer (Fig. 1C). The RNase R assay revealed that circFOXM1 was enriched in U251 and SHG44 cells, and circFOXM1 was more stable than FOXM1 mRNA (Fig. 1D), moreover, circFOXM1 exhibited preferential accumulation in the cytoplasm compared with the nucleus (Fig. 1E). Immunofluorescence confirmed that circFOXM1 was localised in the cytoplasm (Fig. 1F). Expression of circFOXM1 in glioma tissues and glioma cell lines was significantly higher than in normal tissues and control-cell line (HEB), and circFOXM1 in tissues from different grades of glioma indicated that circFOXM1 in high grade glioma (Grade III, IV) was upregulated compared with low-grade glioma (Grade I, II), with U251 and SHG44 cell lines exhibiting the highest levels of circFOXM1 among the glioma-cell lines examined in this study (Fig. 1G, H). The survival curve revealed that the survival rate decreased when the circFOXM1 expression levels were high; in contrast, the survival rate increased when circFOXM1 expression levels were low (Fig. 1I).

### circFOXM1 promotes glioma-cell progression both in vitro and in vivo

**Fig. 2:**
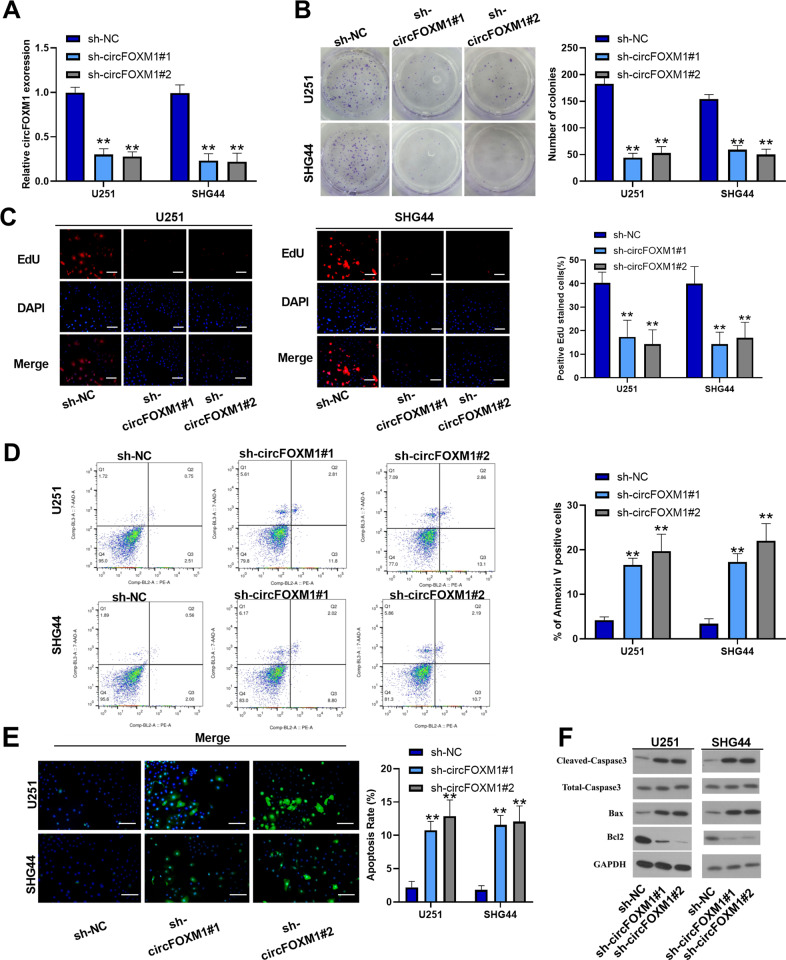
circFOXM1 promotes glioma-cell progression in vitro. **A** The two designed siRNAs sh-circFOXM1#1 and #2 decreased the expression of circFoxM1, demonstrating targeting efficacy. **B**, **C** The proliferation of glioma cells was detected by colony formation and EdU assays in response to knockdown of circFOXM1. **D**, **E** Apoptosis of glioma detected by flow cytometry analysis and TUNEL assay. **F** Western blot was used to measure the expression of apoptosis-related proteins in glioma cells with or without circFOXM1 silencing (**P < 0.01) (scale bar = 100 μm).

To explore the mechanism of circFOXM1 in glioma cells, we selected U251 and SHG44 cell lines to perform the following experiments. Sh-circFOXM1#1 and #2 were established to inhibit the expression of circFOXM1, and qRT-PCR results revealed that sh-circFOXM1#1 and #2 efficiently suppressed the expression of circFOXM1 in both cell lines (Fig. 2A). The colony-formation assay indicated that circFOXM1 downregulation significantly decreased the proliferation of glioma cells (Fig. 2B). EdU staining assays showed that the viability of glioma cells was reduced when circFOXM1 expression was inhibited (Fig. 2C). Flow cytometry (FCM) assayand TUNEL staining assays revealed that the rate of apoptotic cells, which were Annexin-V positive or TUNEL positive, was remarkably increased as circFOXM1 was downregulated (Fig. 2D, E). Western blot analysis demonstrated that expression of Bcl-2 was decreased when circFOXM1 was inhibited, while expression of Bax, cleaved caspase-3, and total caspase-3 was increased. The results also indicated that apoptosis was enhanced when circFOXM1 was downregulated (Fig. 2F). Transwell assays revealed that migration and invasion were attenuated in response to downregulation of circFOXM1 (Fig. S2A, B).

**Fig. 3:**
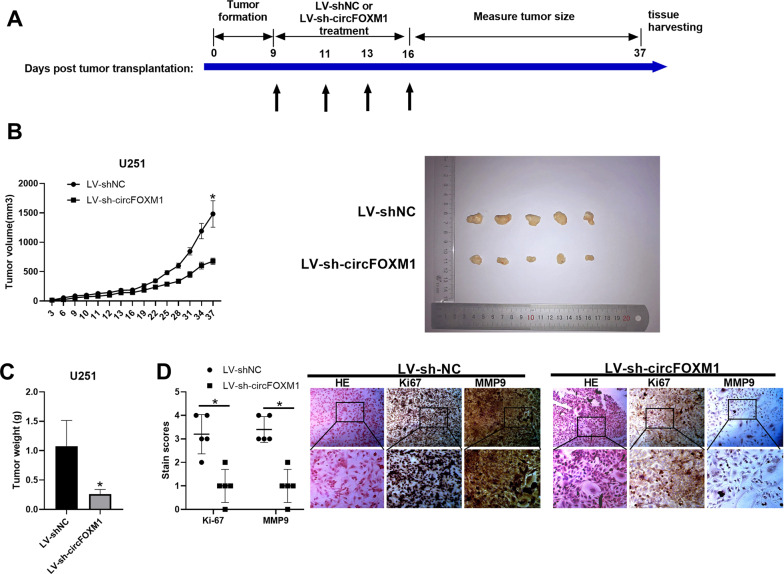
Downregulation of circFOXM1 suppresses glioma progression in vivo. **A** Schematic diagram of the tumour-xenograft experimental process. **B** Tumour volume at different times measured every 3 days. **C** Tumour weight in the LV-sh-circFOXM1 group and LV-sh-control group. **D** IHC staining of Ki-67, MMP9 and EGFR expression in subcutaneous tumours (*P < 0.05) (scale bar = 100 μm).

A nude mouse tumour-xenograft experiment was performed to investigate the mechanism of circFOXM1 in vivo. A total of 1 × 10^8^ U251 cells were subcutaneously injected into nude mice, and LV-sh-circFOXM1 or LV-sh-control was injected into nude mice at 9, 11, 13, and 16 days after tumour cell injection. Then, we measured the tumour volumes every 3 days until 37 days after tumour-cell injection. Mice were subsequently sacrificed, and tumours were isolated for further investigation (Fig. 3A). The volume of tumours 37 days after tumour-cell injection was significantly reduced in response to injection of LV-sh-circFOXM1 (Fig. 3B), and tumour weight was lower in the LV-sh-circFOXM1 group than in the LV-sh-control group (Fig. 3C). Meanwhile, the proliferation marker Ki67 and the invasion and migration marker MMP9 were significantly decreased in the tumour tissues of nude mice (Fig. 3D).

### circFOXM1 sponges miR-432 in glioma cells

**Fig. 4:**
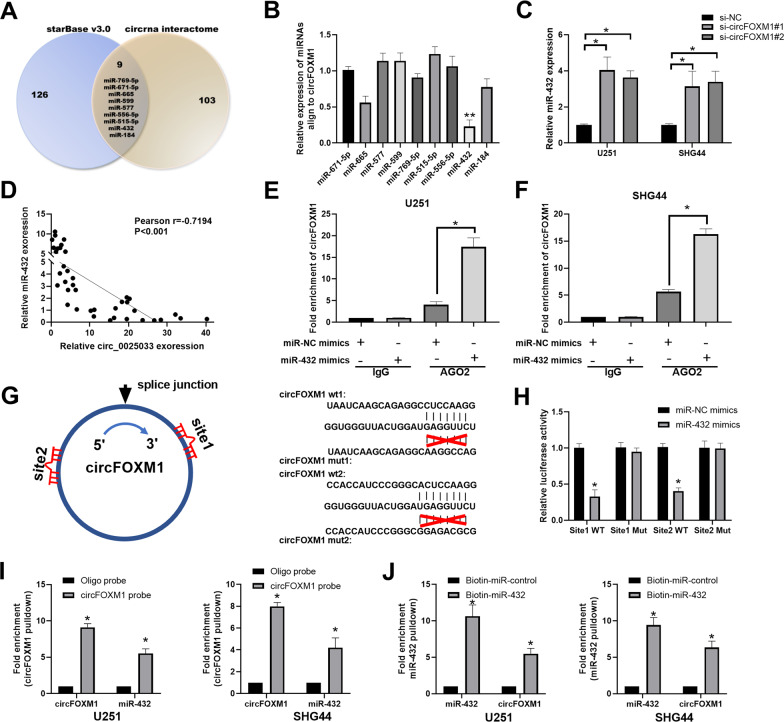
circFOXM1 sponges miR-432 in glioma cells. **A** circFOXM1-binding prediction to miRNAs using two online software programs, StarBase V3.0 and circRNA interactome. **B** Changes in miRNAs were examined in response to overexpression of circFOXM1 in U251 cells. **C** circFOXM1 knockdown significantly increased miR-432 expression. **D** The Pearson correlation between circFOXM1 and miR-432 levels was measured in the same set of glioma tissues. The ΔCt values (normalized to GAPDH) were subjected to Pearson correlation analysis (*R* = −0.7194, *P* < 0.001). **E**, **F** RIP assay to detect the correlation between circFOXM1 and miR-432. **G**, **H** Schematic representation of the potential binding sites between circFOXM1 and miR-432. Luciferase-reporter activity of constructed plasmids (circFOXM1-WT or circFOXM1-MUT) in glioma cells cotransfected with miR-432 mimics or miR-NC mimics. **I**, **J** RNA pull-down assay to detect the correlation between circFOXM1 and miR-432 (*P < 0.05, **P < 0.01).

To explore the mechanism of circFOXM1, we predicted potential miRNAs to which circFOXM1 binds using StarBase V3.0 and the circRNA Interactome online database and found that nine miRNAs have the potential to bind circFOXM1, including miR-769-5p, miR-671-5p, miR-665, miR-599, miR-577, miR-556-5p, miR-515-5p, miR-432, and miR-154 (Fig. 4A). Additionally, we detected the expression of 9 miRNAs in response to overexpression of circFOXM1 in U251 cells using qRT-PCR and found that miR-432 exhibited the greatest reduction (Fig. 4B). Conversely, expression of miR-432 was remarkably increased when circFOXM1 was inhibited in U251 and SHG44 cells (Fig. 4C). To clarify the relationship between circFOXM1 and miR-432, qRT-PCR of glioma tissues (*n* = 40) revealed that circFOXM1 and miR-432 are negatively correlated (Fig. 4D). RIP assay results revealed that circFOXM1 and miR-432 combined with Ago-2 in U251 and SHG44 cells (Fig. 4E, F). The results of the luciferase-reporter assay, which might demonstrate the interaction between circFOXM1 and miR-432, revealed that application of the miR-432 mimic remarkably weakened the luciferase activity of wild-type (WT) circFOXM1 in response to transfection of negative control (NC). However, the miR-432 mimic did not influence the luciferase activity of the circFOXM1 mutant (Fig. 4G, H). RNA pull-down assays indicated that biotin-coupled circFOXM1 successfully pulled down miR-432, and biotin-labelled miR-432 also pulled down circFOXM1 (Fig. 4I, J). Taken together, these results indicate that circFOXM1 acts as a sponge of miR-432 in glioma cells.

### MiR-432 inhibition rescues the effects of circFOXM1 knockdown on glioma cells

**Fig. 5:**
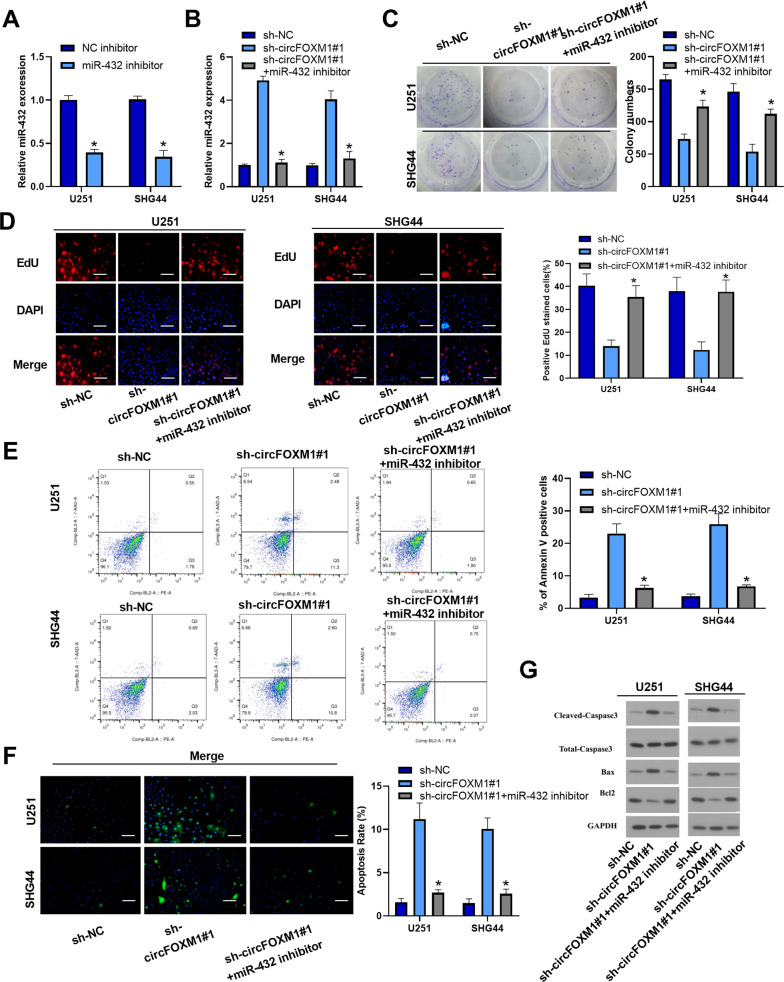
miR-432 inhibition rescues the effects of circFOXM1 knockdown on glioma cells. **A**, **B** MiR-432 knockdown efficiency and its expression in different transfection groups were examined by qRT-PCR in U251 and SHG44 cells. **C**, **D** The proliferation ability of glioma cells was detected by colony formation and EdU assays in response to knockdown of circFOXM1. **E**, **F** Apoptosis of glioma cells detected by flow cytometry analysis and TUNEL assay. **G** Western blot was used to measure the expression of apoptosis-related proteins in glioma cells with or without circFOXM1 silencing (*P < 0.05) (scale bar = 100 μm).

To explore the mechanism of miR-432 in glioma cells, a miR-432 inhibitor was used to perform functional rescue studies, and the qRT-PCR results revealed that the miR-432 inhibitor efficiently suppressed the expression of miR-432 in U251 and SHG44 cells (Fig. 5A), and inhibition of miR-432 reduced the expression of miR-432 when circFOXM1 was knocked down (Fig. 5B). The colony-formation assay revealed that inhibition of miR-432 remarkably increased the proliferation of glioma cells when circFOXM1 was downregulated (Fig. 5C). EdU staining assays revealed that the miR-432 inhibitor increased the viability of glioma cells when the expression of circFOXM1 was inhibited (Fig. 5D). FCM and TUNEL staining assays revealed that inhibition of miR-432 remarkably reduced the rate of apoptotic cells, which were Annexin-V positive or TUNEL positive, when circFOXM1 was downregulated (Fig. 5E, F). Western blot analysis demonstrated that the miR-432 inhibitor increased expression of Bcl-2 when circFOXM1 was inhibited and decreased the expression of Bax, cleaved caspase-3, and total caspase-3 (Fig. 5G). Transwell assays revealed that the miR-432 inhibitor strengthened migration and invasion when circFOXM1 was downregulated (Fig. S3A, B). These findings demonstrate that inhibition of miR-432 reverses the effects of circFOXM1 knockdown in glioma cells.

### CircFOXM1 binds miR-432 to regulate the expression of Gα12 and the RhoA signalling pathway

**Fig. 6:**
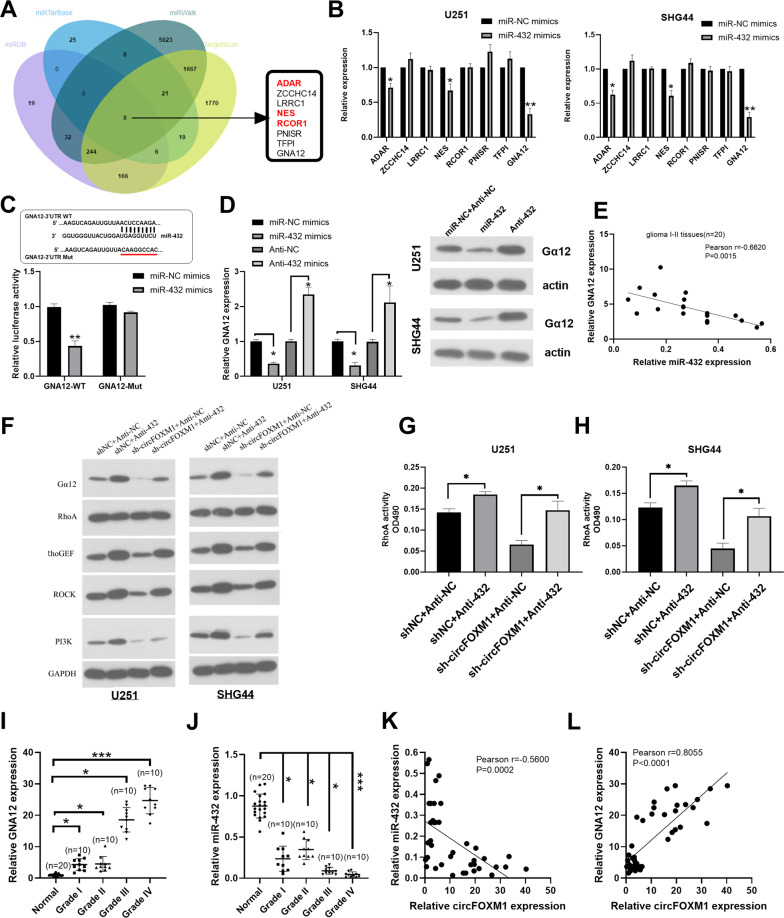
circFOXM1 binds miR-432 to regulate the expression of Gα12 and the RhoA signalling pathway. **A** A total of 8 targeted mRNAs of miR-432 from four online databases. **B** The interrelation between targeted mRNAs and miR-432 was verified by qRT-PCR. **C** Luciferase-reporter activity of constructed plasmids (Gα12-WT or Gα12-MUT) in glioma cells cotransfected with miR-432 mimics or miR-NC mimics. **D** Relative expression of Gα12 in U251 and SHG44 cells transfected with miR-432 mimics or miR-432 inhibitor. **E** The Pearson correlation between Gα12 and miR-432 levels was measured in the same set of glioma tissues. The ΔCt values (normalized to GAPDH) were subjected to Pearson correlation analysis (R = −0.6620, *P* = 0.0015). **F–H** Western blotting was used to measure the expression of the RhoGEF/RhoA/ROCK/PI3K pathway. **I**, **J** The relative expression of Gα12 and miR-432 in glioma tissues of different grades. **K** The Pearson correlation between circFOXM1 and miR-432 levels was measured in the same set of glioma tissues. The ΔCt values (normalized to GAPDH) were subjected to Pearson correlation analysis (*R* = −0.5600, *P* = 0.0002). **L** The Pearson correlation between circFOXM1 and Gα12 levels was measured in the same set of glioma tissues. The ΔCt values (normalized to GAPDH) were subjected to Pearson correlation analysis (*R* = 0.8055, *P* < 0.0001) (*P < 0.05, **P < 0.01).

To further detect the gene playing the primary role in response to miR-432 and CircFOXM1, four online databases were utilized to predict the downstream target gene of miR-432. We screened 8 potential genes; however, ADAR, NES, and RCOR1 have already been reported by other studies (Fig. 6A) Interestingly, qRT-PCR showed that Gα12 exhibited the greatest reduction when miR-432 was overexpressed in glioma cell lines (Fig. 6B). Gα12 was highly regulated in both GBM and LGG tissues (Fig. S4A), and the overall survival and disease-free survival curves indicated that the survival rate was decreased when the expression level of Gα12 was high (Fig. S4B, C). Next, siGα12 was used to clarify the effect of Gα12 in glioma. siGα12 efficiently suppressed the expression of Gα12 in U251 and SHG44 cells (Fig. S4D). The colony formation assay showed that siGα12 remarkably decreased the proliferation of glioma cells (Fig. S4E), and the Transwell assay showed that siGα12 decreased the migration ability of glioma cells (Fig. S4F). Subsequently, we investigated the effect of Gα12 on the circFOXM1 and miR-432 pathways. The results of the luciferase-reporter assay suggested that the miR-432 mimic remarkably weakened the luciferase activity of wild-type (WT) Gα12 compared with transfection of the negative control (NC) (Fig. 6C), and Western blot results demonstrated that expression of Gα12 was increased when miR-432 was inhibited. In contrast, expression of Gα12 was decreased when miR-432 was overexpressed (Fig. 6D). qRT-PCR of glioma tissues (*n* = 20) showed that Gα12 and miR-432 were inversely correlated (Fig. 6E). Western blot analysis of U251 and SHG44 cells indicated that the miR-432 inhibitor increased expression of Gα12 when circFOXM1 was inhibited, as well as the RhoA protein (Fig. 6F–H). Gα12 and miR-432 in tissues from different grades of glioma showed that Gα12 in high-grade glioma (Grade III, IV) was upregulated compared with that in low-grade glioma (Grade I, II); however, miR-432 in high grade glioma (Grade III, IV) was downregulated compared with that in low-grade glioma (Grade I, II) (Fig. 6I, J). qRT-PCR of glioma tissues (*n* = 40) demonstrated that circFOXM1 and miR-432 had an inverse correlation, while circFOXM1 and Gα12 had a positive correlation (Fig. 6K, L). These results revealed that circFOXM1 binds miR-432, which regulates the expression of Gα12 and the RhoA signalling pathway.

### CircFOXM1 promotes tumorigenesis of glioma cells via Gα12

**Fig. 7:**
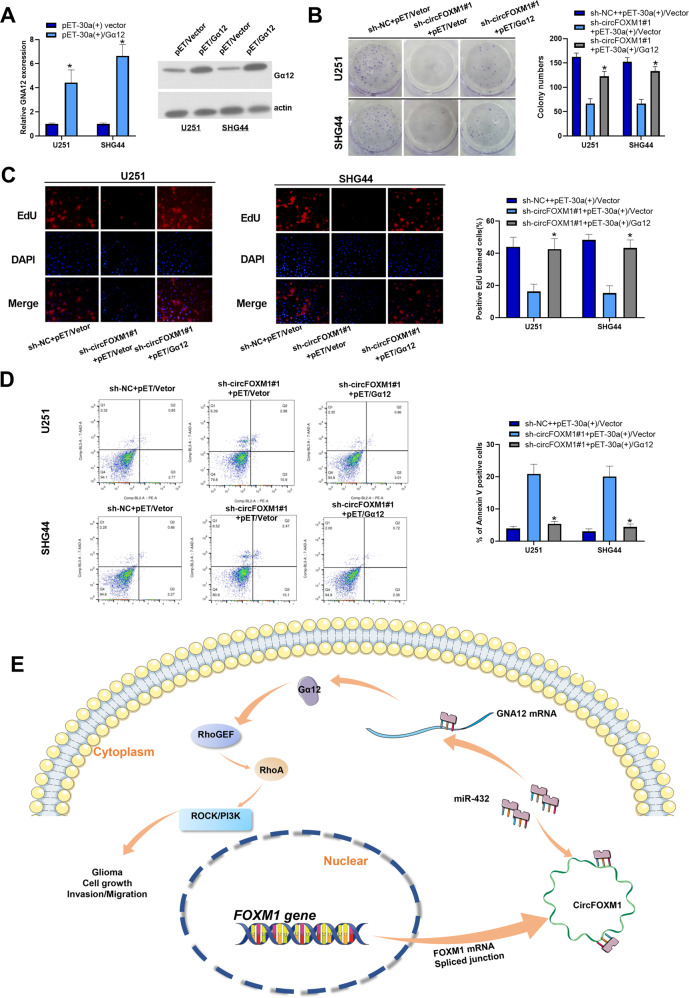
circFOXM1 promotes the tumorigenesis of glioma cells via Gα12. **A** Expression of Gα12 in glioma cells in response to pET-30a(+)/Gα12 transfection. **B**, **C** The proliferation of glioma cells was detected by colony-formation and EdU assays in response to circFOXM1 knockdown. **D** Apoptosis of glioma cells detected by flow cytometry analysis. **E** Schematic illustration of the proposed mechanism of CircFOXM1 in glioma (*P < 0.05).

To verify the effect of Gα12 on circFOXM1 in glioma, Gα12 was overexpressed using pET-30(+)/Gα12. Western blot analysis revealed that pET-30(+)/Gα12 efficiently boosted the expression of miR-432 in U251 and SHG44 cells (Fig. 7A). The colony-formation assay indicated that the overexpression of Gα12 significantly increased the proliferation of glioma cells when circFOXM1 was downregulated (Fig. 7B). EdU staining assays revealed that the overexpression of Gα12 increased the viability of glioma cells when the expression of circFOXM1 was inhibited (Fig. 7C). FCM and TUNEL assays revealed that the overexpression of Gα12 reduced the rate of apoptotic cells, which were Annexin-V positive, when circFOXM1 was downregulated (Fig. 7D, S5A). Transwell assays revealed that the overexpression of Gα12 strengthened migration and invasion when circFOXM1 was downregulated (Fig. S5B, C).

In general, these results indicate that circFOXM1, which originates from FOXM1 mRNA, sponges miR-432, which regulates the expression of Gα12, further influencing the RhoGEF/RhoA/ROCK/PI3K pathway to mediate glioma-cell growth and aggressiveness (Fig. 7E).

## Discussion

In recent years, the rise of bioinformatics in the cancer-research field has facilitated discovery of an increasing number of circRNAs in different cancers that modulate tumorigenesis, metastasis, and chemoresistance [12, 22, 23]. For instance, circHIPK3, a particularly abundant and upregulated circRNA in colorectal cancer cells, might sponge endogenous miR-7 to inhibit miR-7 activity and then increase FAK, IGF1R, EGFR, and YY1 expression to promote colorectal cancer growth and metastasis [24]; circ_0001361 acts as an oncogene in the invasion and metastasis of breast cancer by targeting the miR-491-5p/MMP9 axis [25]; circ-RanGAP1 overexpression is associated with a high TNM stage of gastric cancer; and the circ-RanGAP1 mediates the miR-877-3p/VEGFA axis to promote gastric cancer progression [26]. Noticeably, we found that circFOXM1 was upregulated in glioma tissues and cells and hypothesised that it was linked to the prognosis of glioma patients, necessitating further exploration of its role in glioma.

Glioma is a fatal intracranial tumour that has the characteristics of high morbidity and poor clinical prognosis [27], and surgical treatment and chemoradiotherapy do not satisfactorily promote the overall survival rate of patients [28, 29]. In recent years, some emerging therapies, such as gene therapy, virotherapy and molecular targeted therapy, have achieved some advances in the laboratory-research phase, laying a foundation for the clinical transformation of novel therapies [30–32]. However, these modalities still need additional research to improve quality of life in glioma patients. Therefore, a novel valid diagnostic and therapeutic target is urgently needed.

In this study, we analysed the FOXM1 gene in the UCSC database, screened circFOXM1 (circ_0025033) to further explore its role in glioma. We observed that the expression of circFOXM1 was upregulated in both glioma tissues and cell lines. In addition, we verified that circFOXM1 restrained cell proliferation and activated glioma-cell apoptosis in vitro and inhibited tumour growth in vivo when circFOXM1 is knocked down. Upregulation of circFOXM1 may decrease the survival rate in glioma patients. These data demonstrated that circFOXM1 acts as an oncogene in glioma, and the molecular mechanism needs to be further explored.

The competing endogenous RNA (ceRNA) hypothesis connects protein-coding mRNAs with noncoding RNAs, such as circRNAs, lncRNAs, and miRNAs, ultimately influencing physiological and pathological processes [33–35]. The term “RNA sponge” represents the phenomena in which miRNAs are adsorbed by transcripts and involves overexpressed miRNA response elements (MREs), such as lncRNAs, circRNAs, and pseudogenes [36, 37]. A single miRNA can regulate multiple targets containing a particular MRE of the miRNA, and it has been demonstrated that the miRNA-mediated ceRNA mechanism might be a universal form of post-transcriptional regulation [38]. Moreover, the effect of RNA sponges was discovered in multiple cancers, indicating that ceRNAs play a crucial role in cancer [39, 40].

Additionally, we clarified that circFOXM1 binds to miR-432 using RIP, RNA pull-down, and luciferase-reporter assays. Then, rescue experiments indicated that inhibition of miR-432 rescued the effects of circFOXM1 knockdown in glioma cells. Taken together, circFOXM1 acts as a sponge of miR-432 in glioma cells. The miR-432 downstream gene Gα12 is highly expressed in glioma cells, indicating that Gα12 silencing might limit the progression of glioma. Western blot analysis of glioma cells indicated that miR-432 inhibition increased the expression of Gα12 and the RhoA protein when circFOXM1 was inhibited. These findings reveal that circFOXM1 binds miR-432 to regulate the expression of Gα12 and the RhoA signalling pathway to promote the tumorigenesis of glioma.

Despite these findings, this study has some limitations to consider that should be acknowledged and used to improve future research. First, there were few patients who volunteered to provide glioma tissues to identify the expression of circFOXM1, and we would like to screen additional volunteers to verify circFOXM1 as an oncogene in glioma. Second, additional target genes related to circFOXM1 should be explored to clarify additional pathways that might be associated with glioma.

In summary, we demonstrated that circFOXM1 is substantially upregulated in human glioma tissues and cell lines and that circFOXM1 acts as a sponge of miR-432 to promote the proliferation and aggressiveness of glioma cells. Additionally, we demonstrated that circFOXM1 might promote the tumorigenesis of glioma cells through the Gα12 signalling pathway. circFOXM1 has feasibility as a novel biomarker of glioma diagnosis, and circFOXM1 might represent a novel therapeutic target for glioma in the future.

## Materials and methods

### Clinical samples

Forty glioma-tissue samples and twenty normal brain-tissue samples were recruited from glioma patients who were definitively diagnosed with glioma at Chang Sha Central Hospital between May 2018 and December 2020. All tumour samples were clinicopathologically confirmed as glioma (10 Grade I, 10 Grade II, 10 Grade III, and 10 Grade IV). Normal brain tissues were collected from patients undergoing brain-tissue resection due to craniocerebral injury. All the tissues were stored at −80 °C.

### RNA extraction and PCR

Total RNA was extracted from glioma tissues and cells using TRIzol reagent (Invitrogen, USA) according to the manufacturer’s instructions. Reverted First Strand cDNA Synthesis (TransGen, China) was applied to synthesize cDNA from RNA. qRT-PCR was performed using SYBR Premix Ex Taq II (TransGen, China) in an ABI 7500 PCR instrument. U6 served as the control to standardize circRNA expression. Primers were purchased from GenePharma.

### Plasmids, siRNAs, and transfection

All plasmids, sh-circFOXM1, and miR-432 mimics or inhibitors were provided by GenePharma. Transfection was performed using Lipofectamine 3000 reagent (Invitrogen, USA) according to the manufacturer’s protocol.

### Dual-luciferase reporter assay

Glioma cells (1 × 10^5^/well) were prepared in 12-well plates with 200 ng pGL3/circFOXM1 WT or pGL3/circFOXM1 Mut and 100 nmol miR-432 mimic transfection. After 48 h, the transfected cells were analysed using luciferase activities detected using the Dual-Luciferase Reporter Assay System (Promega, USA).

### Cell migration and invasion assay

Migration and invasion were assessed using Transwell assays. Then, 100 µL of serum-free medium with Matrigel (BD Biosciences) (5:1) was added into the Transwell chamber (0.8 μm, Corning, USA) and incubated at 37 °C for 4 h until the mixture became solid. The cell suspension was prepared at a density of 1 × 10^5^/mL, 100 μL of cell suspension was added to each well in a 24-well plate (Corning, USA), and 500 μL of culture medium with 20% FBS was added to the bottom of the chamber. Methanol was used to immobilize the cells in the membrane, which were subsequently stained with crystal violet. The results were analysed microscopically.

### Colony-formation assay

Transfected U251 and SHG44 cells were utilized 24 h after transfection, and 300 cells/well were inoculated into 6-well plates. After incubation at 37 °C for 14 days, the colonies were fixed in 4% paraformaldehyde and stained with crystal violet solution.

### Statistical analysis

The data are presented as the mean ± standard deviation (SD) and were analysed using one-way analysis of variance and Tukey’s post hoc multiple comparison tests. All data in this study were analysed using SPSS 25.0. Values were considered statistically significant when *P*-values were <0.05.

## Supplementary information

Supplemental meterial

Language Editing Certificate

## Data Availability

All data that support the findings of this study are available from the corresponding authors upon reasonable request.
