# Supplementary material for: The involvement of the circFOXM1–miR–432–Gα12 axis in glioma cell proliferation and aggressiveness
Source: Cell Death Discov. 2022 Jan 10;8:9. doi: 10.1038/s41420-021-00782-9 (PMC8748925; doi:10.1038/s41420-021-00782-9)
Supplement: Supplementary file 1 — Supplemental meterial [file 41420_2021_782_MOESM1_ESM.docx]

**Supplementary** **Materials and Methods**

**Cell culture**

Human glioma cell lines (U373, U251, SHG44, A172, TJ905, LN229) and normal cells (HEB), human papillary thyroid carcinoma cell lines (K1，FTC133), human ovarian cancer cell lines (A2780,SKOV3), human lung cancer cell lines (A549, H299), human colon cancer cell lines (HT-29, SW480), were kindly provided by Cell Bank of Chinese Academy of Sciences. All cells were propagated in high glucose Dulbecco’s Modified Eagle Medium (DMEM, Gibco, USA) containing 10% FBS (Gibco, USA) and stored in an incubator with 5% CO2 at 37 °C.

**Tumor xenograft model**

Male BALB/c nude mice (6-8weeks, 20–30 g) were prepared for the subcutaneous tumor xenograft, and the study in vivo was admitted by the Ethics Committee of Chang Sha Central Hospital. 1 × 10^8^ U251 cells were subcutaneously injected into nude mice, and LV-sh-circFOXM1 or LV-sh-control was been injected into nude mice 9 days after the cell injection. Then we measured the tumor volumes every 3 days, and assessed the volumes by the formula: volume (cm^3^) = (length × width^2^)/ 2. After 37-day of injection, mice were sacrificed and tumors were obtained to further investigation.

**RNA pull-down assay**

U251 or SHG44 cells were granulated and resuspended in RIPA buffer(Thermo Fisher, USA), Oligo circFOXM1 or Biotin-miR-432 was incubated with the cell lysates for 1 h. Afterward, the beads were used to enrich pull-downs and qRT-PCR analysis of indicated RNAs was followed.

**RNA immunoprecipitation (RIP) assay**

1×10^7^ glioma cells lysed in the RIP lysis buffer were prepared for the immunoprecipitation with beads conjugated to antibodies (Millipore, Billerica, MA) against control IgG or human Ago2. Precipitated RNAs were assayed by qRT-PCR.

**TUNEL** **assay**

A terminal deoxynucleotidyl transferase-mediated dUTP nick end labeling assay (TUNEL; Roche, Basel, Switzerland) was used to detect glioma cells apoptosis according to the manufacturer’s instructions. Images were captured by fluorescence microscope (Leica, Solms, Germany). Apoptotic neuronal cells were determined by counting the number of total TUNEL- and DAPI-stained cells.

**5-Ethynyl-2′-deoxyuridine (EdU) assay**

EdU assay kit (Abcam, USA) was used to detected DNA synthesis and cell proliferation. 1×10^4^ glioma cells were seeded in a 96-well plate overnight, and then EdU solution (25 μM) was added into the 96-well plate and incubated for 24 h. Afterwards, 4% paraformaldehyde was applied to fix the cells at RT for 30min. 0.5% TritonX-100 was used to permeabilize the cells for 10 mins and then added Apollo reaction solution (200 μL) to stain the EdU for 30 mins and Hoechst 33342 (200 μL) to stain the nuclei. Finally, we visualized under a fluorescent microscope (Olympus, Japan) to observe DNA synthesis and cell proliferation.

**Flow cytometry (FCM) assay**

Glioma cells were cultured in 24-well plates and incubated at 37℃ for 12h, and then rinsed by 1 ×PBS at least 3 times. The cells were resuspended with 250μl Binding Buffer, and took 100 μL cell suspension into 5 mL flow tube, added 5 μL Annexin V-PE and 10 μL 7-AAD solution (Solarbio, China). The apoptosis rate of glioma cells was measured by the FACSCalibur flow cytometer (BD Biosciences).

**Western blotting**

Protein lysates were extracted from U251 and SHG44 cells by incubating with RIPA lysis and extraction buffer (Thermo Fisher, USA). Protein concentration was detected by BCA kit (Thermo Fisher, USA). Equal amounts of proteins were separated by sodium dodecyl sulfate polyacrylamide gel electrophoresis, transferred to polyvinylidene difluoride membranes (Millipore, USA), and incubated overnight at 4°C with primary antibodies followed by blocking with bovine serum albumin (5%, v/v). The primary antibodies included Bax (1:1000, rabbit IgG; Abcam, USA), Bcl-2 (1:1000, rabbit IgG; Abcam, USA), cleaved caspase-3 (1:2000, rabbit IgG; Abcam, USA), total caspase-3 (1:1000, Abcam, USA), Gα12 (1:2000, rabbit IgG; Abcam, USA), RhoA(1:1000, rabbit IgG; Abcam, USA), RhoGEF(1:1000, rabbit IgG; Abcam, USA), ROCK(1:1000, rabbit IgG; Abcam, USA), PI3K(1:1000, rabbit IgG; Abcam, USA), GAPDH(1:1000, rabbit IgG; Abcam, USA). Membranes were then incubated with the secondary antibody (1:4000, Proteintech, China) for 120 min at RT. Reacting bands were visualized using enhanced chemiluminescence reagent (Proteintech, China) and the density of the protein bands was semi-quantified using the software Image J.

**Supplementary Figures**


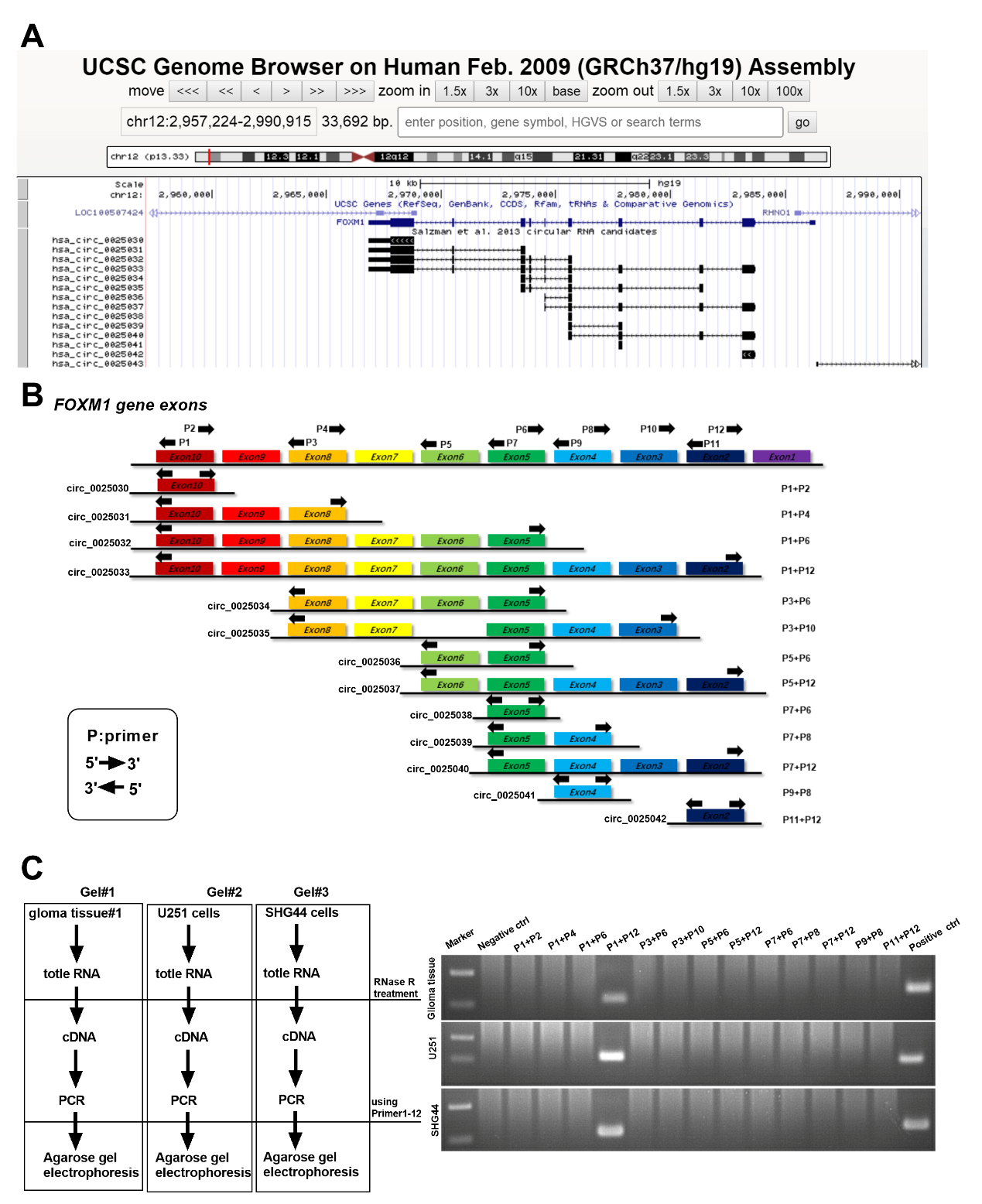


**Figure S1. Identification of circFOXM1. A)** FOXM1 gene was analyzed in UCSC database, and discovered that the exons encode 13 circRNAs; **B-C)** 12 primers have been established to measure the expression of 13 circRNAs in glioma cell lines and tissues.


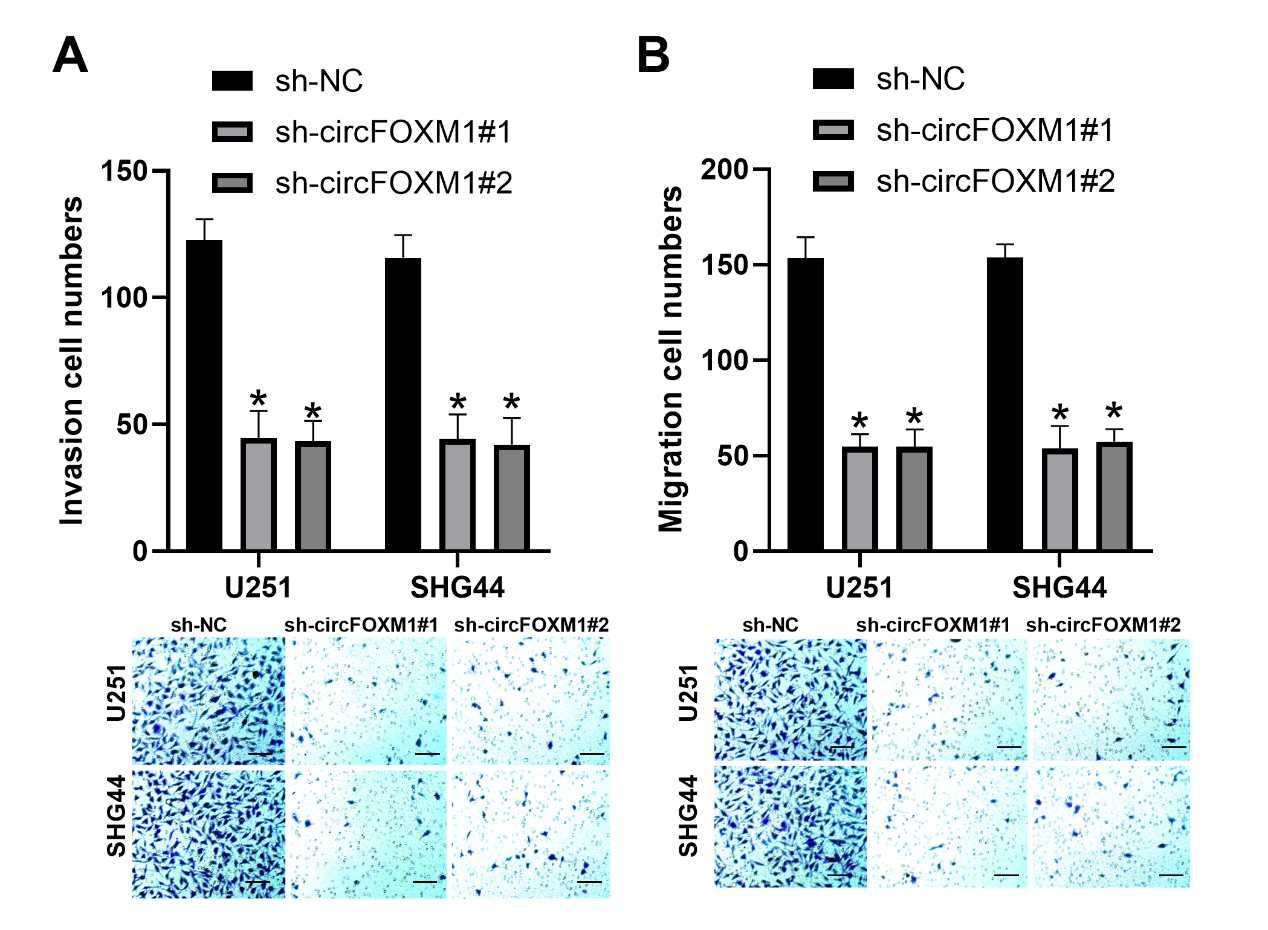


**Figure S2. Invasion and migration assay of glioma cells. A)** The ability of invasion of glioma cells were detected by transwell assay when knocking down circFOXM1; **B)** The ability of migration of glioma cells were detected by transwell assay when knocking down circFOXM1. (**P* < 0.05) (scale bar = 100 μm)


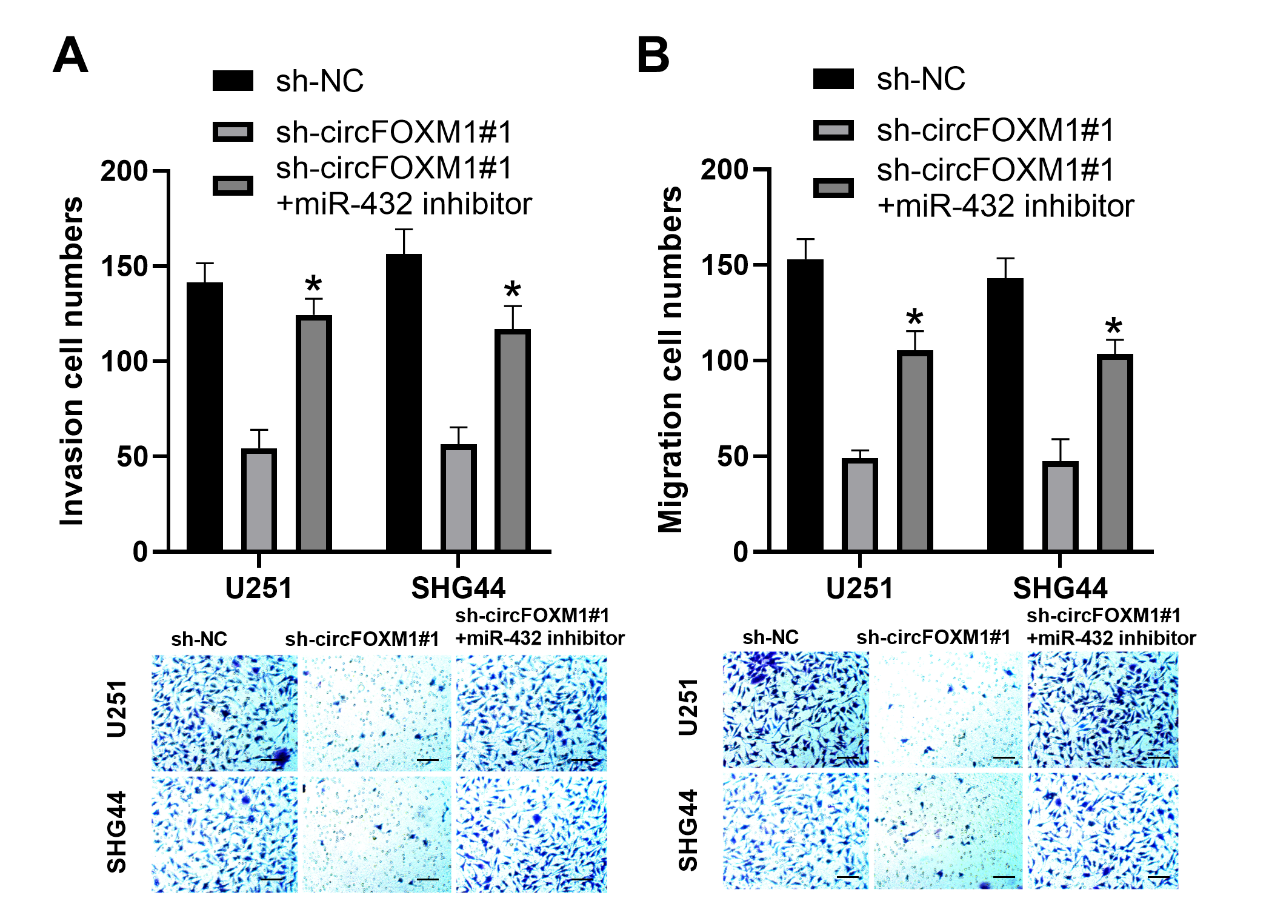


**Figure S3. Invasion and migration assay of glioma cells. A)** The ability of invasion of glioma cells were detected by transwell assay when transfecting the miR-432 inhibitor and knocking down circFOXM1; **B)** The ability of migration of glioma cells were detected by transwell assay when transfecting the miR-432 inhibitor knocking down circFOXM1. (**P* < 0.05) (scale bar = 100 μm)


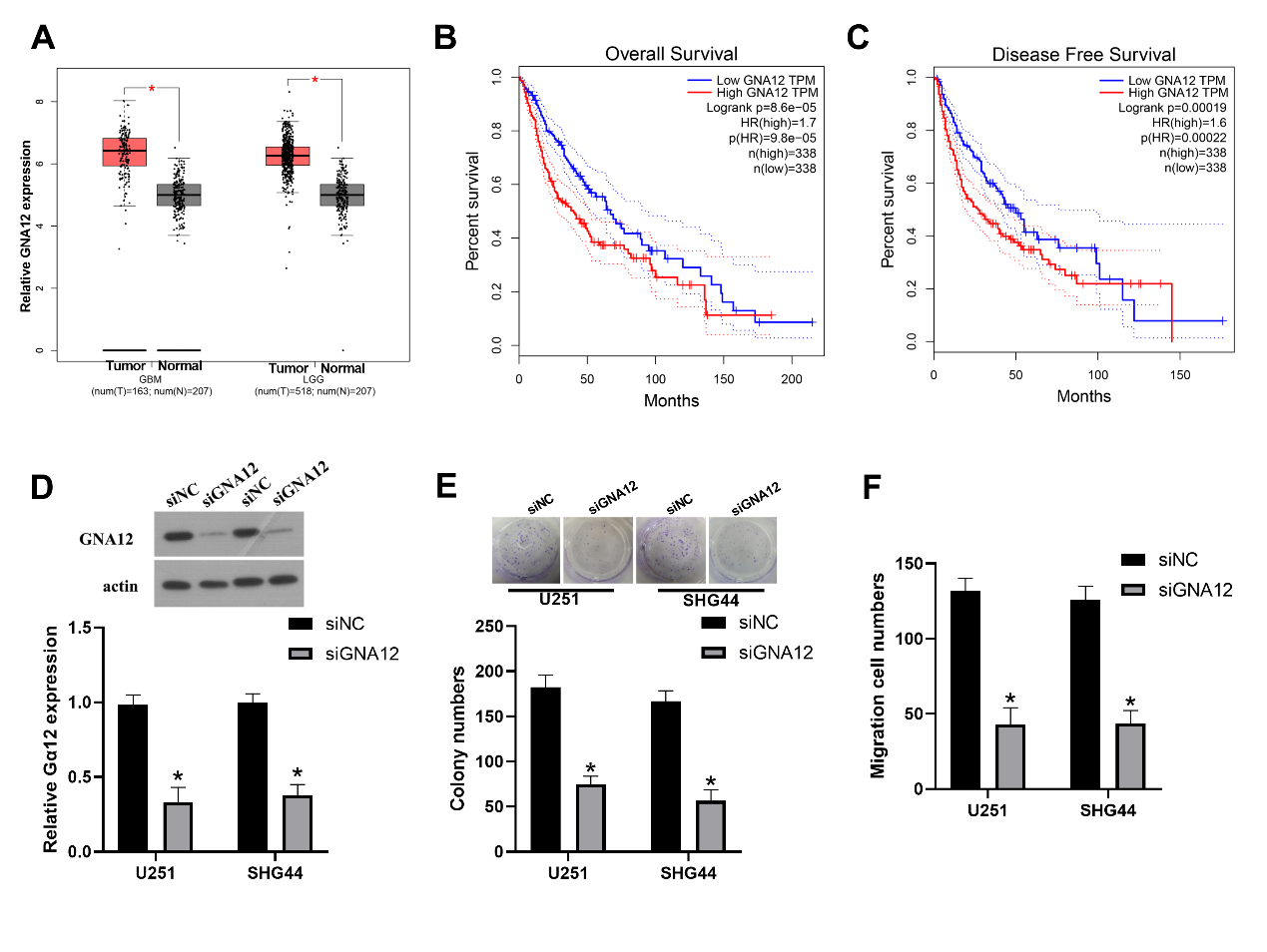
**Figure S4. CircFOXM1 decoys miR-432 to regulate the expression of Gα12 and RhoA signaling pathway. A) The expression of** Gα12 was measured by qRT-PCR in both GBM and LGG tissues; **B-C)** the overall survival and disease free survival curves of the patients of the expression level of Gα12 was high of low; **D)** siGα12 was efficiently suppress the expression of Gα12 in U251 and SHG44 cells; **E)** The colony formation assay was performed to detect the proliferation of glioma cells; **F)** the transwell assay was performed to detect the ability of migration of glioma cells**.** (**P* < 0.05)


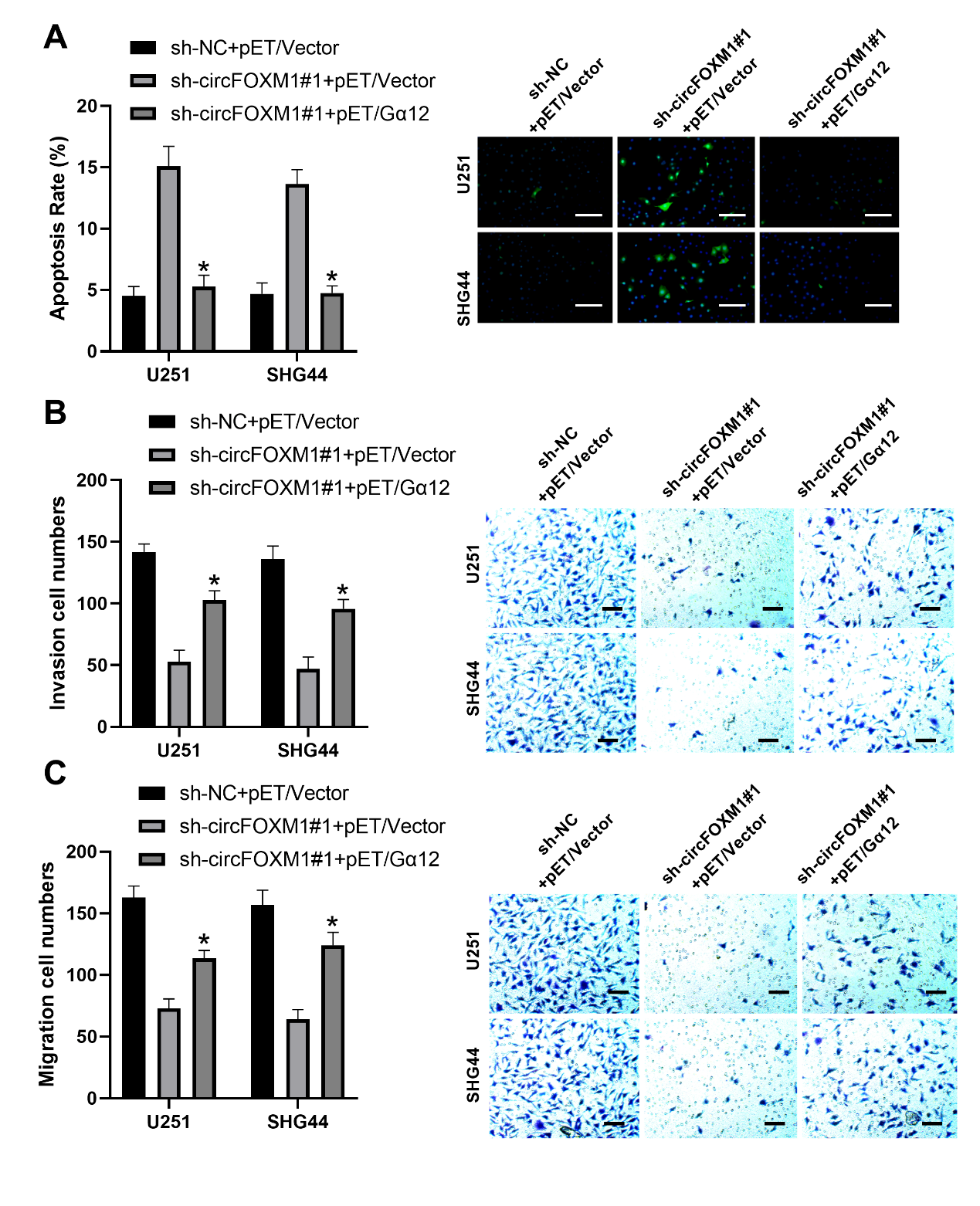


**Figure S5. CircFOXM1promotes the tumorigenesis of glioma cells via Gα12. A)** Cell apoptosis of glioma detected by TUNEL assay; **B)** The ability of invasion of glioma cells were detected by transwell assay when knocking down circFOXM1; **C)** The ability of migration of glioma cells were detected by transwell assay when knocking down circFOXM1. (**P* < 0.05) (scale bar = 100 μm).
