# Supplementary material for: The involvement of the circFOXM1–miR–432–Gα12 axis in glioma cell proliferation and aggressiveness
Source: Cell Death Discov. 2022 Jan 10;8:9. doi: 10.1038/s41420-021-00782-9 (PMC8748925; doi:10.1038/s41420-021-00782-9)
Supplement: Supplementary file 2 — Language Editing Certificate [file 41420_2021_782_MOESM2_ESM.pdf]

# Certificate of Elsevier Language Editing Services

The following article was edited by Elsevier Language Editing Services:

"The involvement of the circFOXM1-miR-432-Gα12 axis in glioma cell proliferation and aggressiveness"

Authored by:

Tianyu Fan

Date: 14-Aug-2021
Serial number: LE-218534-6A00CC955EF6
